# Mechanical Characterization and Computational Analysis of TPU 60A: Integrating Experimental Testing and Simulation for Performance Optimization

**DOI:** 10.3390/ma18020240

**Published:** 2025-01-08

**Authors:** Luan Lang, Rodrigo Antunes, Thiago Assis Dutra, Martim Lima de Aguiar, Nuno Pereira, Pedro Dinis Gaspar

**Affiliations:** 1 Department of Electromechanical Engineering, University of Beira Interior, Rua Marquês de D’Ávila e Bolama, 6201-001 Covilhã, Portugal; luan.lang@ubi.pt (L.L.); rodrigo.antunes@ubi.pt (R.A.); thiago.dutra@ubi.pt (T.A.D.); martim.aguiar@ubi.pt (M.L.d.A.); nuno.pereira@ubi.pt (N.P.); 2 C-MAST—Centre for Mechanical and Aerospace Science and Technologies, 6201-001 Covilhã, Portugal

**Keywords:** TPU 60A, material characterization, tensile tests, printing orientations, simulation validation, mechanical properties, ISO 37, ISO 527, additive manufacturing

## Abstract

This study investigates the mechanical properties of thermoplastic polyurethane (TPU) 60A, which is a flexible material that can be used to produce soft robotic grippers using additive manufacturing. Tensile tests were conducted under ISO 37 and ISO 527 standards to assess the effects of different printing orientations (0°, 45°, −45°, 90°, and quasi-isotropic) and test speeds (2 mm/min, 20 mm/min, and 200 mm/min) on the material’s performance. While the printing orientations at 0° and quasi-isotropic provided similar performance, the quasi-isotropic orientation demonstrated the most balanced mechanical behavior, establishing it as the optimal choice for robust and predictable performance, particularly for computational simulations. TPU 60A’s flexibility further emphasizes its suitability for handling delicate objects in industrial and agricultural applications, where damage prevention is critical. Computational simulations using the finite element method were conducted. To verify the accuracy of the models, a comparison was made between the average stresses of the tensile test and the computational predictions. The relative errors of force and displacement are lower than 5%. So, the constitutive model can accurately represent the material’s mechanical behavior, making it suitable for computational simulations with this material. The analysis of strain rates provided valuable insights into optimizing production processes for enhanced mechanical strength. The study highlights the importance of tailored printing parameters to achieve mechanical uniformity, suggesting improvements such as biaxial testing and G-code optimization for variable thickness deposition. Overall, the research study offers comprehensive guidelines for future design and manufacturing techniques in soft robotics.

## 1. Introduction

Due to technological developments in recent decades, the use of robots and the automation of processes has become trivial, from simple tasks to highly complex operations [1]. Robots have become relevant equipment in factories, providing the opportunity to replace 4D (dull, dirty, dangerous, and dear) tasks performed by humans, implementing improvements, and reducing costs in production processes [2].

One of the robot’s possible end effectors is a gripper, for grasping and manipulating objects. Grippers, like robots themselves, are fundamental elements in industry, since manipulating objects with various shapes is one of the most complex challenges in robotics [3]. To handle sensitive products or those with complex geometries, soft grippers made from flexible materials have been developed, allowing considerable deformation to adjust to the product without damaging it.

These grippers allow for superior results when manipulating sensitive and irregular objects, compared to rigid grippers [4]. Until now, the prototyping of soft grippers was predominantly carried out through manual processes, such as silicone molding, due to the lack of accessible and developed technologies for production. However, advances in additive manufacturing and flexible materials technologies have significantly enhanced the customization, adaptability, and complexity of soft grippers. While additive manufacturing is not the sole factor making soft grippers viable, it plays a key role in enabling their tailored designs and broader application. These advancements have made soft grippers an increasingly practical and cost-effective solution for complex tasks, such as safe human interaction and handling fragile objects, by offering flexible and innovative design possibilities [5].

The fragility, sensitivity, stickiness, and slipperiness of most food and agricultural products have driven the development of soft robotic grippers that are deformable, flexible, safe, low-cost, and environmentally friendly. Such a model of robotic gripper has the potential to meet these challenges by offering reliable and adaptable solutions for handling agricultural and food products [6], since the main application of the soft gripper is to hold and move objects safely without causing significant deformation or damage to their delicate surfaces [7].

Fused Filament Fabrication (FFF) is a popular additive manufacturing technique that has gained significant relevance [8,9]. According to Sukindar et al. [9], FFF was initially developed to produce and create prototypes. Nowadays, it is adopted in different fields, such as architecture, manufacturing, and medical applications. FFF is a type of additive manufacturing process that uses thermoplastic materials to produce three-dimensional objects. The fundamental principle behind FFF involves the controlled extrusion of a thermoplastic filament through a heated nozzle.

Antunes et al. [10,11] focused on optimizing Fin Ray Effect (FRE)-inspired grippers to enhance their adaptability and grasping strength. This design is optimal for production using additive manufacturing, namely Fused Filament Fabrication (FFF). They employed computational design and simulation to improve the performance of FRE soft robotic fingers, and compared two FRE-based soft robotic fingers, utilizing Ansys software to assess how different designs impact gripping efficacy.

The selection of TPU 60A for this study is justified by its marked advantages over comparable materials, such as TPU 95A. TPU 60A exhibits superior elasticity and softness, characteristics that make it particularly well-suited for applications demanding delicate handling, such as soft robotic grippers designed for interaction with fragile or irregularly shaped objects. In contrast, TPU 95A, while offering greater strength and rigidity, may apply excessive pressure, thereby increasing the risk of damage in such sensitive applications. This comparison highlights the appropriateness of TPU 60A for soft robotics, where flexibility and adaptability are critical, and aligns the material selection with the study’s objective of optimizing performance in specialized contexts.

The use of Thermoplastic Polyurethane (TPU) 60A in soft robotics requires an understanding of its mechanical behavior under various conditions. However, the existing literature and supplier-provided data lack comprehensive experimental characterization of TPU 60A across different test speeds and printing orientations. There is an absence of data detailing how varying the test speeds and orientations affect the material’s mechanical properties, such as elongation, strength, and stress–strain response. This gap hinders the development of accurate constitutive models for computational simulations. To address this gap, an experimental investigation of TPU 60A was conducted. Tensile tests were performed at three test speeds (2 mm/min, 20 mm/min, and 200 mm/min) and four printing orientations (0°, 45°/−45°, 90°, and quasi-isotropic configurations). By systematically analyzing the effects of these variables, a detailed characterization of the material’s mechanical properties is provided, which is necessary for predictive modeling and simulation purposes.

The experimental data reveal the influence of different test speeds and printing orientations on the mechanical behavior of TPU 60A, including the identification of ideal stress–strain curves for each configuration. For constitutive modeling in simulations, the most suitable model to represent TPU 60A’s behavior was determined. This model can be used in simulations, using software such as Ansys (version 2023 R1), which will be used to simulate the mechanical characteristics of soft grippers made in TPU 60A. By inputting the experimentally obtained stress–strain data into Ansys, the software’s curve-fitting tool was used to generate the required parameters for the selected constitutive model. This ensured that the simulated material behavior closely matched the experimental results. Providing these detailed parameters enables accurate replication of the material’s response in computational analyses, easing the adoption of TPU 60A in various engineering applications where simulations closely mirror real-world performance.

The key contribution of this work is the development and validation of a constitutive model for TPU 60A that represents its mechanical behavior when testing different test speeds and orientations. Integrating this model into Ansys software allowed for performing numerical simulations of soft grippers made from TPU 60A, and comparing the simulation results with experimental tensile test data demonstrated that the model predicts material performance, with relative errors between simulated and experimental results less than 5%, confirming the model’s validity. By providing empirical data and validated constitutive models, this work explores the design and optimization of devices using TPU 60A. Simulating the material’s behavior fastens the development process, reducing the need for extensive physical prototyping and enabling more efficient iterations. In addition, the authors also believe that this research work contributes to the field of soft robotics—particularly to soft grippers—where material properties play a critical role in the functionality and effectiveness of devices.

## 2. Materials and Methods

Following the recommendations of the datasheet for Filaflex TPU 60A of the manufacturer Recreus. Elda, Spain [12], the 3D printer must be properly configured, since the material has a shore hardness of 60A, which is very elastic with a stretch ability of 950%.

It is important to note that the manufacturer does not provide specific details regarding the exact printing conditions or methods used to achieve this value. The only information available indicates that the tests were conducted following the DIN 53504-S2 standard [13]. This lack of detailed guidance may explain differences in results, regarding factors such as printing parameters (e.g., nozzle temperature, infill density, or print speed), potential imperfections introduced during the printing process (e.g., voids or layer adhesion issues), and deviations in test conditions (e.g., strain rates or environmental factors) that can significantly impact mechanical properties. Furthermore, the use of additive manufacturing introduces anisotropic properties, which could also contribute to the observed variations in elongation at break.

In the present work, the 3D printer Artillery Sidewinder X1 (Artillery 3D Technology Co., Ltd., Shenzhen, China) was used to manufacture the specimens. It has characteristics that, within its market range, facilitate the printing of flexible polymers, such as TPU. Primarily, a direct extrusion system and polytetrafluoroethylene (PTFE)-lined hotend allow for a straightforward printing of flexible thermoplastics [14].

The tensile tests were carried out using a Shimadzu Autograph AGS-X-50 kN high-performance universal testing machine (Shimadzu Group, Kyoto, Japan). It has a maximum force capacity of 50 kN, an effective test width of 425 mm, and a maximum crosshead displacement of 745 mm. It is equipped with TRAPEZIUM LITE X software (version: 1.0) to control tests in real-time and to acquire data [15].

The open-source software UltiMaker Cura (version 5.0) [16], a Computer-Aided Manufacturing (CAM) software, was used to convert the 3D models into the G-code that guides the printing process. Table 1 presents the printing parameters adopted in the present work.

The specimens were designed using SolidWorks software according to ISO 37 and ISO 527 standards [17,18], which were also followed when carrying out the tensile tests.

To investigate the time-dependent mechanical response of TPU 60A, the specimens were tested at three different crosshead test speeds (v): 2 mm/min, 20 mm/min, and 200 mm/min. To verify the influence of printing orientations, four sample models were printed, each containing eight specimens, meaning a total of 32 specimens. The samples were printed in four different orientations: 0° (along the Y direction), 45°, and −45°, 90° (along the X direction), and a quasi-isotropic configuration covering the orientations of 0°, 45°, −45°, and 90°. This procedure was based on studies in the literature, such as those by Naveed et al. [19] and Frenkel et al. [20], who investigated different printing orientations to improve the mechanical behavior of specimens.

**Figure 1:**
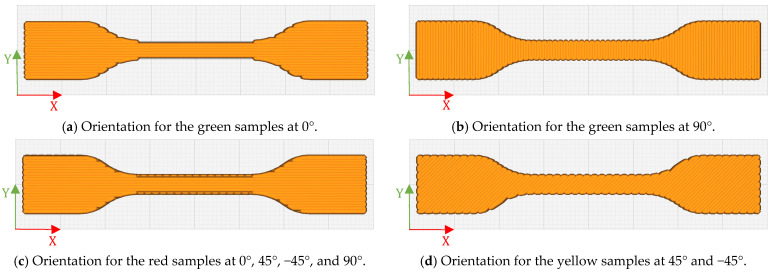
Orientation for the sample ISO 37: (**a**) at 0°; (**b**) at 90°; (**c**) at 0°, 45°, −45°, and 90°; (**d**) at 45° and −45°.

**Figure 2:**
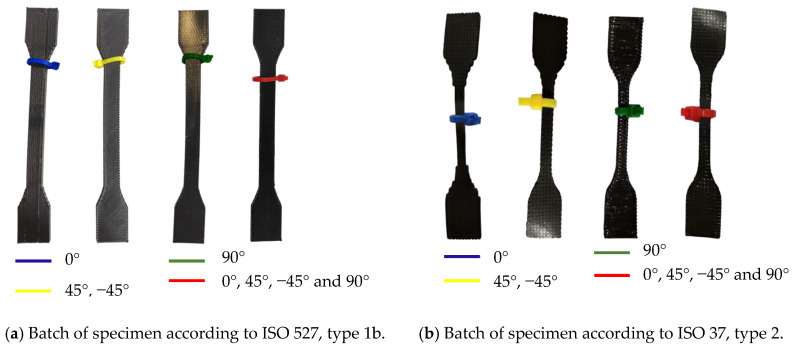
Batch of printed samples categorized by color for different printing orientations: (**a**) ISO 527, type 1b.; (**b**) ISO 37, type 2.

To comply with ISO 527 and ISO 37 standards, five specimens from each set of eight were considered valid, respecting the requirement to test a minimum of five specimens for each of the established test orientations [17,18], as seen in Figure 1, where it shows the four different orientations in the software Ultimaker Cura. The variation in printing orientation allowed for the analysis of how this variable affects the quality and accuracy of tensile tests, offering a more detailed and representative view of the material’s performance. This helps ensure that the material meets the specifications and requirements of technical standards. To facilitate differentiation, the orientations were categorized by color: blue for the longitudinal [0°], green for the transverse [90°] orientations, red for the quasi-isotropic orientation [0°, 45°, −45°, 90°], and yellow for the [45°, −45°] orientation, as shown in Figure 2. These four printing models follow the definitions in Table 1.

Table 2 summarizes the number of valid specimens and respective test speeds tested for each orientation.

**Figure 3:**
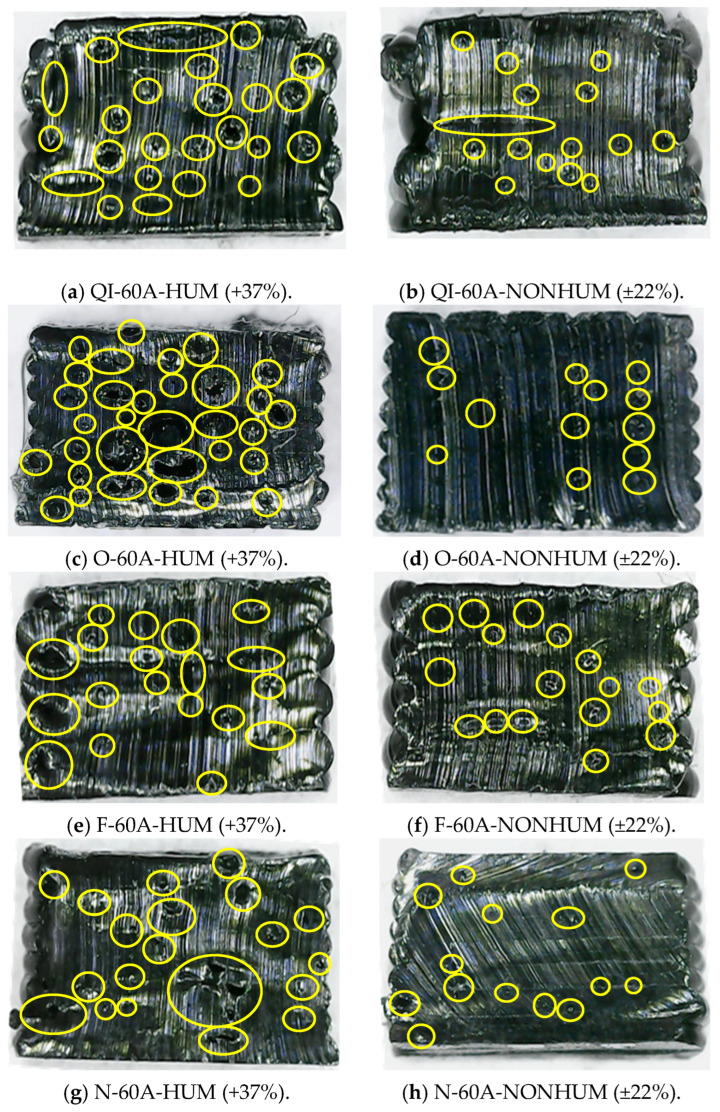
Cross-section area of the specimens with high and low humidity levels.

The presence of voids in the printed specimens also influences their mechanical properties, leading to premature failure and reduced stiffness. Even though TPU 60A is an elastomeric material, the voids formed during the printing process compromise its resistance. Cross-sectional analysis, as shown in Figure 3, reveals that specimens printed with high-humidity filament exhibit a greater number of voids compared to those printed with low-humidity filament. These voids act as stress concentrators, weakening the material and causing it to fail at lower strains than expected.

The cross-sectional analysis of the specimens shown in Figure 3 reveals the presence of gaps and voids formed during the printing process. Specimens printed with filament at higher humidity levels (+37%) exhibit a significantly greater number of voids compared to those printed with low humidity filament (±22%). This analysis demonstrates a correlation between filament humidity and void formation in the printed structures. Specifically, samples with higher humidity (Figure 3a,c,e,g) contain markedly more voids and gaps within their microstructure than their dehumidified counterparts (Figure 3b,d,f,h).

The percentage of the void area within the samples was quantified using ImageJ software (version 1.54i) [21], which provides an accurate and efficient method for analyzing particles and void distributions in image data. Table 3 presents a comparison of void percentages for various samples under different humidity conditions, specifically highlighting the significant impact of filament humidity levels on void formation.

Effective control of filament humidity and printing conditions is critical for minimizing void formation and enhancing the mechanical integrity and reliability of printed elastomeric structures. Proper humidity management reduces void density, resulting in improved durability and more consistent mechanical performance. These findings highlight the critical role of filament preparation processes in additive manufacturing, particularly for materials like TPU 60A, and provide practical insights for optimizing production workflows. By ensuring reduced void formation, the material’s performance becomes more reliable for applications requiring precise mechanical properties, such as soft robotics and other specialized fields.

The results reveal a consistent trend: samples printed with higher humidity levels (+37%) exhibit significantly greater void percentages compared to their low-humidity counterparts (±22%). For instance, the quasi-isotropic configuration (QI-60A-HUM) exhibits a void percentage of 12.13%, which is more than four times higher than its low-humidity counterpart, QI-60A-NONHUM, at 2.80%. Similarly, the 0° orientation (O-60A-HUM) shows the highest void percentage among all samples (24.90%), compared to only 3.87% for its low-humidity equivalent. Similar patterns are observed for the 45°/−45° (F-60A) and 90° (N-60A) orientations, where high-humidity samples have void percentages of 9.94% and 15.12%, respectively, compared to 3.76% and 2.98% in their low-humidity counterparts.

The increased void density in high-humidity specimens can be attributed to moisture absorption by the filament. During extrusion, the absorbed moisture evaporates, creating bubbles that lead to voids within the material. These voids compromise the overall density and mechanical integrity of the printed samples. Conversely, samples printed with lower humidity levels demonstrate significantly fewer voids, reinforcing the importance of proper filament drying and controlled environmental conditions in reducing void formation.

With this analysis, this test highlights the importance of dehumidifying filament both before and during the 3D printing process. Dehumidification reduces moisture content, thereby minimizing void formation and ensuring more uniform and dense material deposition. By eliminating these defects, the mechanical performance of printed components is significantly enhanced, resulting in increased strength, superior surface finish, and greater reliability. This is particularly important for applications that demand precise and consistent material properties.

Thus, specifically for this work, the TPU 60A filament was dried using a desiccant dryer, and the filament was kept inside the dryer throughout the printing process to maintain the lowest possible humidity levels. During the tests, the ambient humidity level was controlled at approximately 22%.

## 3. Results

The experimental characterization of tensile mechanical properties was carried out according to ISO 37 and ISO 527 standards [17,18]. During the tests, the data were acquired at a rate of 1 Hz for a crosshead test speed (v) of 2 mm/min, and at a rate of 10 Hz for crosshead displacements of 20 mm/min and 200 mm/min. The data collected during the tests were post-processed using routines developed for this study. For the sake of further reproducibility by third parties, these routines are available on GitHub [22].

### 3.1. Printing Orientation Quasi-Isotropic

**Figure 4:**
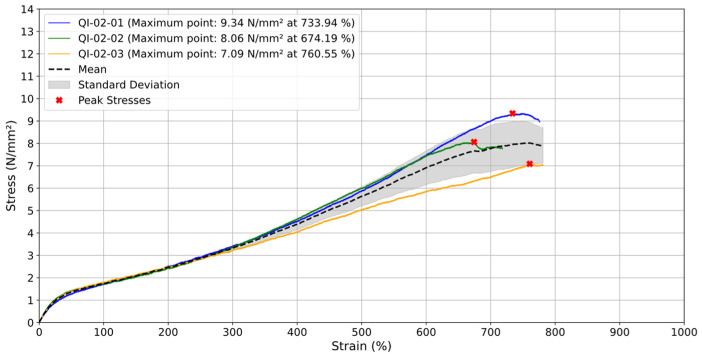
Tensile engineering stress–strain curves for quasi-isotropic samples tested at 2 mm/min.

First, three quasi-isotropic specimens, i.e., those with layers printed at [0°/45°/−45°/90°], were tested until failure at a test speed (v) of 2 mm/min. The engineering stress–strain curves obtained throughout the tests are presented in Figure 4, with the peak stresses indicated by the red markers. From Figure 4, it can be verified a good consistency between the mechanical response until the specimens reach strain levels of 300%. From this point, small deviations are noticed, which can be attributed to imperfections in the specimens that were induced by the manufacturing process. After testing the specimens at a test speed (v) of 2 mm/min, considerable stress relaxation effects were observed, which directly affect the mechanical response of the material. For this reason, this test speed was not adopted throughout the other tests.

Additionally, the stress–strain analysis of quasi-isotropic TPU 60A specimens tested at a test speed (v) of 20 mm/min reveals consistent mechanical behavior during the elastic phase, with near-linear stress increases up to around 600% strain and minimal standard deviation, indicating effective load distribution within the material’s internal structure. Beyond this point, the material enters the plastic regime, where notable variability emerges among the samples. Maximum stress values range from 7.39 N/mm^2^ (QI-20-01) to 8.52 N/mm^2^ (QI-20-04), with differences in strain at rupture, suggesting the presence of internal defects like voids or micro-cracks, likely introduced during the 3D printing process. QI-20-04 and QI-20-05, exhibiting the highest stress and strain values, indicate a more robust material structure, while earlier ruptures in QI-20-01 and QI-20-02 point to potential flaws in printing conditions.

**Figure 5:**
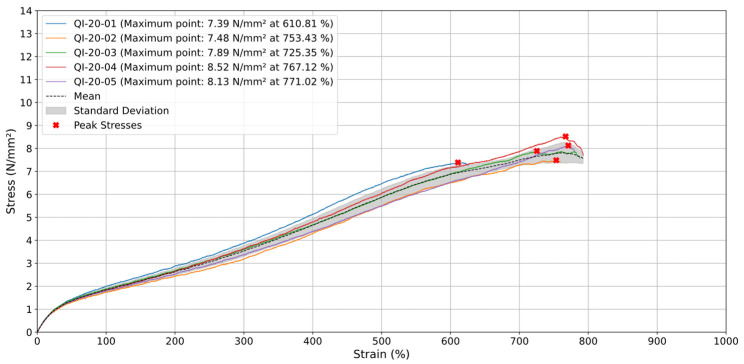
Stress (N/mm^2^) vs. Strain (%) for quasi-isotropic samples at 20 mm/min.

The average results for the quasi-isotropic specimens at 20 mm/min are shown in Figure 5, with stress increasing steadily up to approximately 7.81 N/mm^2^ around 700% strain. The standard deviation remains low throughout most of the test but increases slightly near maximum stress values, reflecting greater variability as the material reaches its deformation limit. In this analysis, QI-20-01 shows a consistent stress increase up to the point of maximum deformation at around 600% strain. QI-20-02 and QI-20-03 achieve higher maximum stress values, suggesting enhanced material strength before rupture. QI-20-04 and QI-20-05 stand out with the highest stress and strain values, indicating a more robust material structure.

**Figure 6:**
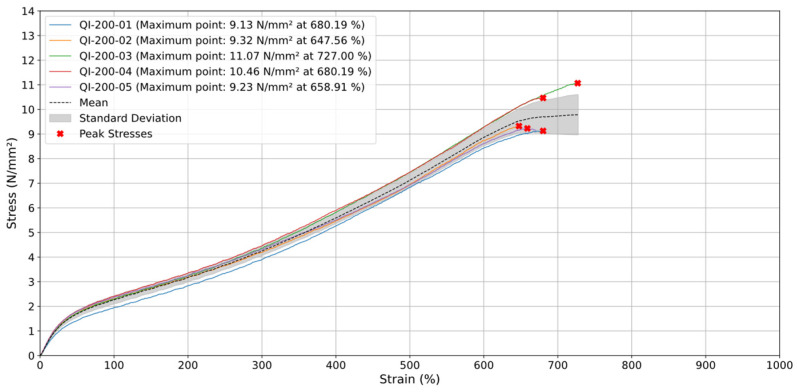
Stress (N/mm^2^) vs. Strain (%) for quasi-isotropic samples at 200 mm/min.

The stress–strain analysis for tests conducted at a test speed (v) of 200 mm/min, shown in Figure 6, reveals higher stress levels achieved at lower strain intervals compared to the 20 mm/min tests, indicating the influence of viscoelastic effects at higher testing speeds. The maximum average stress reached approximately 9.78 N/mm^2^ at around 727% strain. The standard deviation initially remains low but starts to increase beyond 600% strain, similar to the trend observed in the previous test, indicating greater dispersion of stress values as the deformation nears its upper limit.

The results for this orientation exhibit consistent performance, with most rupture points occurring between 600% and 700% strain, except for one sample breaking between 700% and 800%.

**Figure 7:**
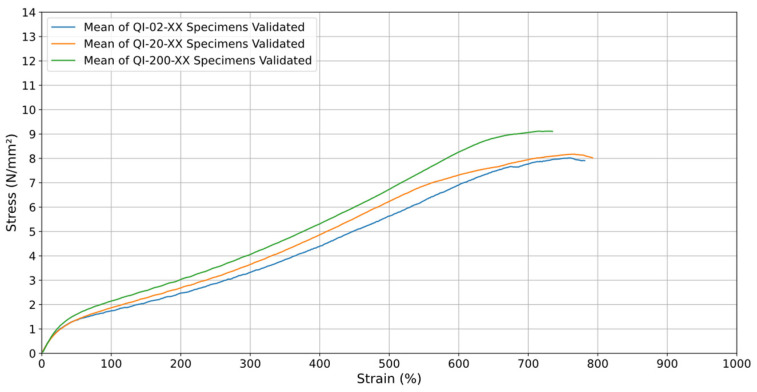
Stress (N/mm^2^) vs. Strain (%) for the averages of the quasi-isotropic specimens at 2 mm/min (blue), 20 mm/min (orange), and 200 mm/min (green).

The analysis across all test speeds highlights the significant impact of testing speed on tensile strength which can be seen in Figure 7. The green curve, representing the 200 mm/min rate, shows a higher maximum stress compared to the orange curve (20 mm/min) and the blue curve (2 mm/min), illustrating that lower test speeds result in lower stress. For all test speeds, the material displays a consistent increase in stress up to about 600% strain, followed by a gradual decrease in the stress growth rate as the deformation progresses. Beyond 700–800% strain, the stress values begin to decline, marking the onset of specimen failure.

The tests conducted at a rate (v) of 200 mm/min resulted in the highest stresses throughout the test, which suggests that increasing the traction speed can increase the mechanical resistance of TPU 60A. This can be explained by the fact that at higher test speeds, the material has less time to accommodate plastic deformation, resulting in more elastic and resistant behavior. In the case of the (v) of 2 mm/min test speed, on the other hand, it led to a more moderate response, with the material reaching lower stress levels for the same deformation. This may be related to a higher relaxation rate of the material when subjected to a lower loading rate, allowing TPU 60A to deform in a more controlled manner and therefore generate lower stresses. For this reason, the tests in terms of test speed (v) of 20 mm/min show an excellent balance between the two test speeds tested.

The mechanical performance of TPU 60A is strongly influenced by testing speed, as shown in Figure 7, across the three strain rates. At higher strain rates, the material demonstrates increased stiffness, enabling robotic grippers to handle heavier loads and resist sudden impacts, which is essential for tasks requiring strength and durability. Conversely, at lower strain rates, TPU 60A retains sufficient flexibility, making it suitable for the delicate handling of fragile or irregularly shaped objects without causing damage. This balance between stiffness and flexibility is particularly important for optimizing robotic gripper performance across a wide range of applications. The viscoelastic properties of TPU 60A allow designers to adapt gripper designs to specific requirements, such as accommodating varying loading speeds or ensuring precise manipulation of sensitive items. By tailoring gripper configurations to leverage these properties, the material’s efficiency and adaptability in industrial, agricultural, and other specialized settings are significantly enhanced. These insights underscore TPU 60A’s versatility and its critical role in advancing soft robotics technologies.

### 3.2. Printing Orientation 0°

**Figure 8:**
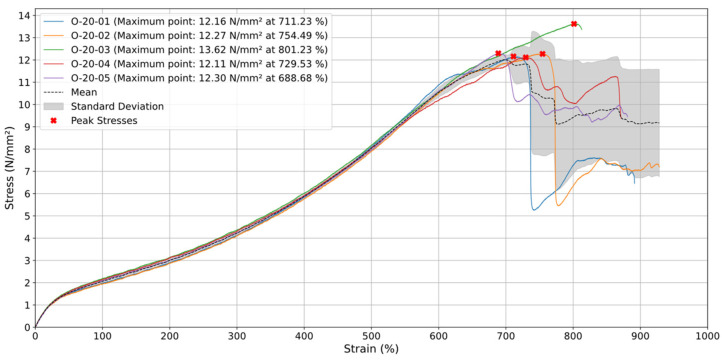
Stress (N/mm^2^) vs. Strain (%) for specimens with orientation of 0° at 20 mm/min.

For the specimens printed at a 0° orientation and tested at a test speed (v) of 20 mm/min, the average stress curve, seen in Figure 8, follows a relatively constant upward trend until around 700% deformation, where the stress reaches its maximum value close to 12 N/mm^2^. This indicates that the material has an elastic and linear behavior during the initial deformation phase. After the maximum stress point, there is a significant decrease in its value, with sharp drops, suggesting a phase of partial rupture and progressive failure of the material, where some strands of deposited material or internal layers may have begun to break down.

In terms of behavior after the stress peak, i.e., after 700% deformation, the curve shows a series of fluctuations, with abrupt drops and subsequent increases, which may indicate the rupture of different layers of material. During this phase, the material still retains some of its structural integrity, but the fluctuations indicate intermittent failures, probably due to the orientation of the layers in relation to traction. In addition, the standard deviation, represented by the shaded area, increases significantly after the point of maximum tension, suggesting greater variability between the specimens tested. This phenomenon can be attributed to the increased fragility of the material as deformation progresses, causing the internal layers to fail unevenly. The presence of voids within the specimen collaborates with this non-uniform failure, further compromising the material’s integrity, such as what happened with the tests O-20-01 and O-20-02.

Along the 0° orientation the printed layers are aligned to the direction of the load, and it is then expected to make the material stronger to a certain extent, but also more susceptible to failure due to delamination between layers. Regarding the final deformation phase occurring after 700%, the sharp drops indicate a widespread material failure, where multiple rupture points seem to occur in sequence. The stress curve tends to drop sharply, and the standard deviation expands further, indicating the unpredictability of the mechanical behavior at this extreme deformation stage. The post-peak behavior reveals the gradual rupture of the deposited material strands, with the material continuing to give way under the load until complete failure.

**Figure 9:**
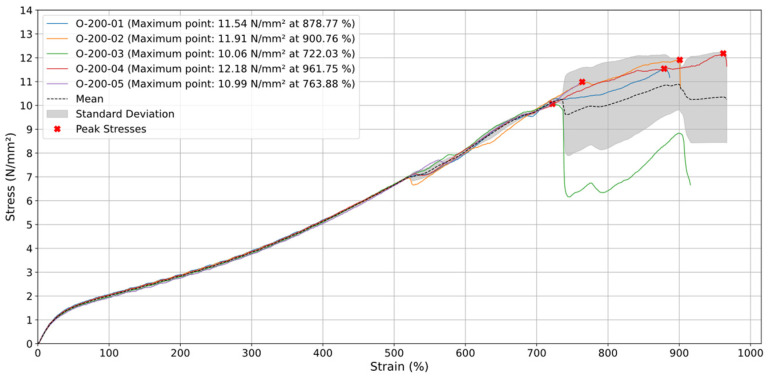
Stress (N/mm^2^) vs. Strain (%) for specimens with orientation of 0° at 200 mm/min.

Figure 9 shows that the 0° specimens subjected to a test speed (v) of 200 mm/min exhibit predictable elastic behavior up to around 700% strain, with stress close to 10 N/mm^2^. A very similar range of results obtained with the 0° specimens tested at a test speed (v) of 20 mm/min.

After this point, the material enters a progressive failure phase, with fluctuations in stress and a considerable increase in standard deviation, reaching a maximum stress of 10.89 N/mm^2^. This suggests variability between the specimens regarding failure behavior. For example, in specimen O-200-03, a minor failure occurs between 700% and 800% deformation, after which the test continues until complete failure is reached at 900% deformation. This happens because the deviation observed in sample O-200-03 likely stems from early, partial failures within its deposited material strands. The higher test speed seems to contribute to a slightly better tensile performance of the material, although the final failure phase continues to show significant variations in the mechanical response of the specimens.

In this case, the localized, partial failures in the specimen’s deposited material strands can dominate the expected viscoelastic response. Typically, lower speeds allow for more time-dependent deformation and relaxation, which would reduce the overall stress. However, when certain strands fail prematurely, the load redistributes irregularly, forcing the remaining intact strands to carry more stress and exhibit more “elastic-like” behavior. This disruption masks the usual viscoelastic effects and results in unexpectedly higher stress values at lower testing speeds.

It is also worth noting that, in these cases, despite the partial rupture of the deposited material strands of the specimens indicated in the graphs by the drops in stress values, the test continues until the final rupture of all the deposited material strands.

### 3.3. Printing Orientation 45° and −45°

**Figure 10:**
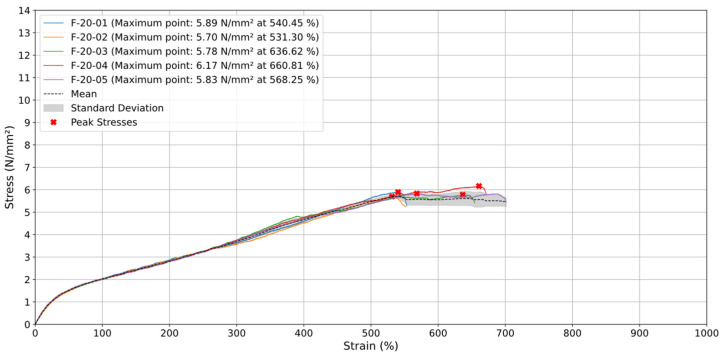
Stress (N/mm^2^) vs. Strain (%) for specimens with orientation of 45° and −45° at 20 mm/min.

In tests involving specimens oriented at 45° and −45° with a test speed (v) of 20 mm/min (Figure 10), failure occurred in two different zones: specimens F-20-01, F-20-02, and F-20-05 broke between 500% and 600% strain. F-20-03 and F-20-04 ruptured between 600% and 700%, and once after exceeding 700% strain. Throughout these tests, stress values remained relatively constant, ranging from 5.7 to 6.17 N/mm^2^. The average stress curve exhibited a linear increase up to approximately 550% strain, reaching a peak of around 5.72 N/mm^2^, followed by a stabilization phase where stress levels stayed between 5 and 6 N/mm^2^ up to 700% strain. The low standard deviation indicates high consistency among the specimens. Unlike 0° orientations, the 45° and −45° specimens did not display a sharp stress drop after reaching maximum stress. Instead, the inclined orientation of the material strands allowed for a more gradual load distribution and controlled deformation, resulting in sustained strength after the stress peak.

**Figure 11:**
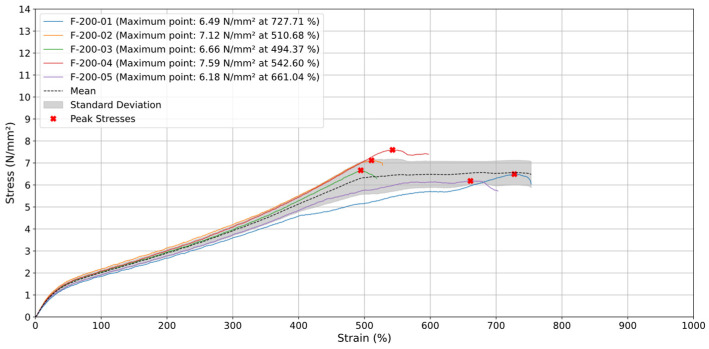
Stress (N/mm^2^) vs. Strain (%) for specimens with orientation of 45° and −45° at 200 mm/min.

When tested at a higher test speed (v) of 200 mm/min (Figure 11), three specimens (F-200-02, F-200-03 (around 500%), and F-200-04) ruptured between 500% and 600% strain with stress values between 6.6 N/mm^2^ and 7.59 N/mm^2^. The remaining two specimens broke after 700% strain, exhibiting a significant decrease in maximum stress to 5.83 N/mm^2^ and 6.17 N/mm^2^, respectively. This behavior highlights the influence of strain rate on the failure characteristics of 45° and −45°-oriented specimens, showing their consistent performance at lower rates and variable outcomes at higher rates.

Additionally, the average stress curve increased linearly up to approximately 500% strain, reaching a peak of around 7 N/mm^2^. Beyond this point, the stress stabilized, fluctuating slightly between 6 and 7 N/mm^2^ until the test concluded at around 750% strain. Similar to tests conducted at a lower test speed of 20 mm/min, the 45° and −45° specimens did not exhibit an abrupt stress drop after reaching maximum stress. Instead, the material maintained its tensile resistance, indicating controlled deformation. This sustained strength suggests that the layer orientation facilitates even load distribution during the final deformation phase, ensuring stability and preventing sudden failure.

The specimens showed high consistency with minimal variability up to around 400% strain. The 45° and −45° orientations were less resistant than 0° due to misalignment with the tensile direction, leading to earlier failure. Testing at a higher test speed of 200 mm/min slightly increased maximum stress and resulted in a smoother stress stabilization phase, indicating improved load management and reduced risk of abrupt failure compared to a lower rate of 20 mm/min.

### 3.4. Printing Orientation 90°

**Figure 12:**
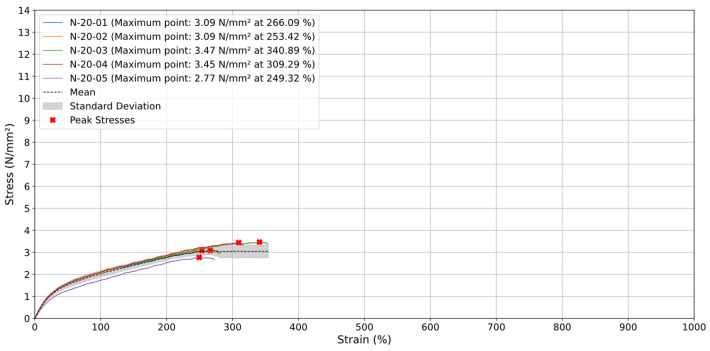
Stress (N/mm^2^) vs. Strain (%) for specimens with orientation of 90° at 20 mm/min.

The specimens printed at 90° tested at a strain rate of 20 mm/min shown in Figure 12 ruptured at lower strain percentages due to the perpendicular orientation of their material strands relative to the applied load. This alignment reduces their tensile resistance, leading to earlier failure compared to specimens with other printing orientations. The delamination between layers, caused by this perpendicular alignment, significantly limits the specimens’ ability to withstand higher stress and strain levels. Specifically, specimens N-20-01, N-20-02, and N-20-05 failed between 200% and 300% strain, while N-20-03 and N-20-04 ruptured between 300% and 400% strain. This result indicates that rupture occurs more quickly under these conditions. In addition to reaching lower maximum stresses, ranging between 2.77 N/mm^2^ and 3.47 N/mm^2^, this happens precisely because the deposited material strands of the specimens are oriented in the opposite direction to the test, resulting in less resistance and leading these specimens to break earlier than the others.

When analyzing the average results for specimens printed at a 90° orientation, the stress–strain curve exhibits a linear increase up to approximately 250% strain, reaching a maximum stress of about 3.09 N/mm^2^. Beyond this point, the stress stabilizes at around 3 N/mm^2^, indicating a zone where the material cannot sustain further strain increases with corresponding stress growth. The standard deviation remains low and consistent throughout most of the test, demonstrating uniform behavior among specimens, with only slight variability in the later stages.

Compared to other orientations (0°, 45°, and −45°), the 90° specimens exhibit significantly lower strain capacity. This reduced performance is attributed to the perpendicular alignment of the printed layers relative to the tensile direction, which promotes delamination and layer separation, thereby limiting the material’s ability to withstand stress and strain.

**Figure 13:**
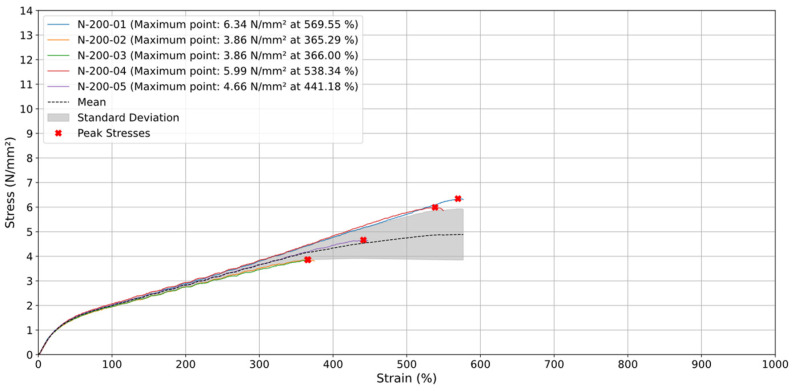
Stress (N/mm^2^) vs. Strain (%) for specimens with orientation of 90° at 200 mm/min.

For specimens printed at 90° and tested at a higher test speed (v) of 200 mm/min (Figure 13), two specimens (N-200-01 and N-200-04) failed between 500% and 600% strain with stress values ranging from 5.99 N/mm^2^ to 6.34 N/mm^2^, while the remaining specimens N-200-02, N-200-03, and N-200-05 broke between 300% and 500% strain. The average stress curve maintained a linear trend up to approximately 500% strain, reaching nearly 5 N/mm^2^, indicating consistent elastic behavior. Unlike tests conducted at 20 mm/min, the higher tensile speed enhanced the material’s tensile strength, allowing strains up to over 550%. However, the standard deviation increased significantly after 400% strain, reflecting greater variability due to layer separation. Despite the higher test speed improving stress resistance, the 90° specimens still demonstrated limited strain capacity and increased vulnerability to failure compared to other orientations.

Testing at a higher test speed of 200 mm/min increased maximum stress and strain capacity slightly but also introduced greater variability in results. Overall, the 90° orientation compromises material strength and deformation capacity, especially under higher strain rates.

Based on the graphs presented in Figure 12 and Figure 13, it can be observed that the 90°-oriented specimens face an additional challenge, as the print orientation is perpendicular to the direction of the applied load during the tensile tests. This results in lower structural resistance since the printed layers are arranged perpendicular to the axis of force, making the specimens more susceptible to failure due to delamination or rupture between layers, compared to different orientations. The anti-parallel layer arrangement directly affects the ability to withstand higher stress, as evidenced by the test results.

## 4. Discussion

### 4.1. Selection of Printing Orientation

**Figure 14:**
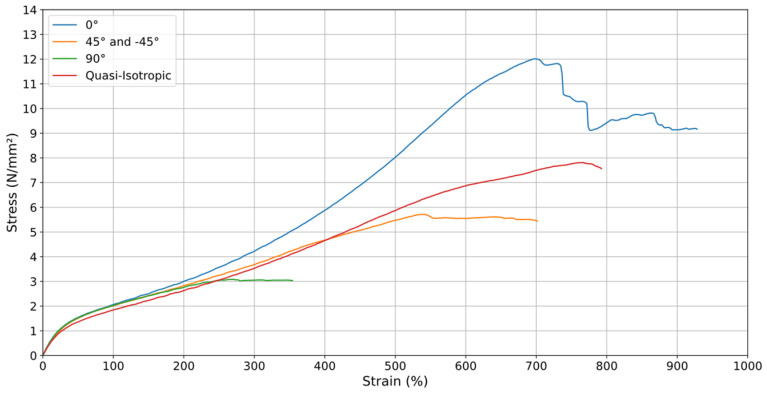
Stress–Strain of printing orientations at (v) of 20 mm/min.

**Figure 15:**
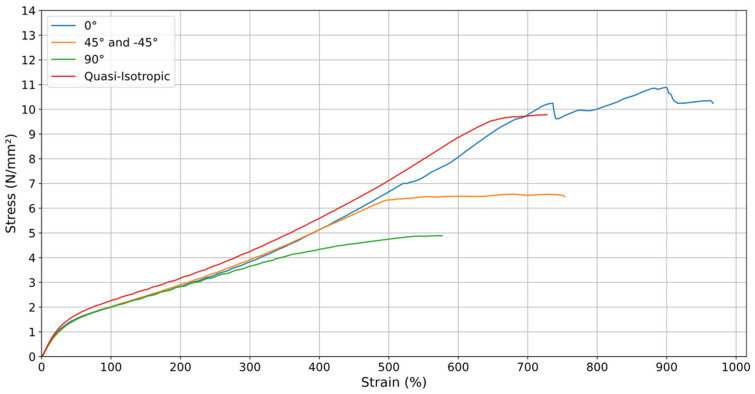
Stress–Strain of printing orientations at (v) of 200 mm/min.

When analyzing the graphs of all orientations at v = 20 mm/min, the orientation that withstands the greatest stress × strain is the orientation when the material was printed at 0°. However, this is not a uniform relationship, as throughout the process, the deposited material strands were breaking, as can be seen in the fluctuations that occur in this sample. Additionally, since the orientation aligns with the machine’s process (vertically), the specimen withstood the stress and continued to perform until the total rupture of its deposited material strands. It is worth noting that the quasi-isotropic orientation, after the 0° orientation, shows the best results at v = 20 mm/min, and it is also the best orientation at v = 200 mm/min (Figure 14 and Figure 15), in addition to being a more uniform curve and thus providing greater reliability in its results. This orientation also showed a smoother and more predictable stress–strain response, indicating a state of greater “equilibrium”. It better represents the 3D printing process, as it incorporates multiple printing directions, effectively distributing stress more uniformly across the material. These qualities make quasi-isotropic orientation more suitable for practical applications and for use in computational simulations where consistency and predictability are critical.

Therefore, it was selected to be used in the computational simulations performed in the Ansys software.

Analyzing Figure 14 and Figure 15, it is evident that the printing orientations significantly influence the material’s mechanical behavior. To enhance uniformity, the top and bottom layers of the specimens, which were originally aligned parallel to the printing bed in a 0° orientation, were removed during the printing process. These layers often have a distinct infill pattern and density compared to the rest of the specimen, potentially leading to variations in mechanical properties. Removing them ensures a more consistent structure and allows for a more accurate assessment of the material’s performance.

By removing these layers, the uniformity of the material’s structure is enhanced, as the remaining infill and layers maintain consistent orientation and density throughout the specimen. This approach ensures that the mechanical behavior observed during testing is not influenced by the additional stiffness or strength contributed by the top and bottom layers, allowing for a more accurate evaluation of the material’s anisotropic properties and its response to tensile loading.

There may also have been manufacturing process failures, such as:

1. Material accumulation or shortage in certain areas during sample fabrication, especially in regions with higher concentrations of localized stresses.
2. Variations in the stiffness of the specimens.
3. Batches of specimens printed on different days.
4. The printers, being frequently used, may have experienced undesirable configuration changes, resulting in slack that causes variations and affects the fabrication of the specimens.

The orientation for the quasi-isotropic TPU 60A specimens was chosen with the objective of obtaining more predictable and consistent material behavior up to rupture. When analyzing the stress vs. strain graphs, the 0° orientation showed greater resistance up to around 700% strain, reaching the highest maximum stress. However, the rupture that occurred after this point was not uniform, with sharp drops and visible fluctuations in the curve, which indicates gradual and less controlled failures in the material.

On the other hand, the quasi-isotropic orientation showed more uniform behavior throughout the test. Despite withstanding slightly lower stresses than the 0° orientation, the material showed a smoother and more continuous curve, with a clean and less abrupt rupture. This suggests that the material distributes the stress better in different directions, resulting in a more predictable and controlled failure. The consistency of the quasi-isotropic curve also indicates greater material reliability in this orientation, which is important for applications where failure prediction and material integrity are essential.

Therefore, the quasi-isotropic orientation was chosen for the simulations as it offers a more stable and reliable representation of the behavior of TPU 60A up to rupture, without the fluctuations observed in the 0° orientation, which can compromise the accuracy of the results and performance analysis in critical applications.

The quasi-isotropic orientation, identified as the most balanced and predictable configuration in this study, offers valuable insights for real-world applications in soft robotics. Its ability to uniformly distribute stress across multiple directions enhances the reliability and adaptability of grippers in handling tasks, particularly those involving irregularly shaped or fragile objects. This characteristic is crucial for applications where mechanical failure could result in damage to sensitive items or inefficiencies in automated systems. Additionally, the uniform mechanical behavior associated with the quasi-isotropic orientation minimizes stress concentrations, which could optimize material usage by reducing wear and extending the operational lifespan of soft robotic components.

In industrial and agricultural settings, the benefits of the quasi-isotropic orientation are particularly pronounced. For example, soft robotic grippers designed with this configuration can provide the strength required for tasks like automated harvesting of delicate produce, such as fruits or vegetables, while maintaining the flexibility needed to adapt to varying shapes and textures. Similarly, in assembly lines, grippers configured with quasi-isotropic orientations can handle sensitive items without compromising precision or causing damage, thus ensuring both efficiency and product integrity.

Furthermore, the findings from this study highlight the potential for TPU 60A, when paired with quasi-isotropic printing, to drive innovations in soft robotics. By offering a reliable and versatile material configuration, these results can guide the development of more robust and adaptable gripper designs tailored to specific applications. This integration of experimental data and practical applications highlights the critical role of material and orientation optimization in advancing the field of soft robotics.

Despite TPU 60A being an elastomeric material, practical tensile tests have confirmed that the printing orientation greatly affects its mechanical performance. The experimental results showed significant variations in tensile strength and maximum elongation based on the layer deposition direction during additive manufacturing. This indicates the material’s inherent anisotropy when fabricated via FFF, requiring consideration of printing orientation in the design and practical use of TPU 60A components.

### 4.2. Computational Simulation Validation

For computational simulations, the finite element method was used. In the context of the numerical simulations performed in the commercial software Ansys [23], the equilibrium equations are applied to each finite element that makes up the mesh of the model. The software solves these equations at each node of the mesh, ensuring that the system is in static or dynamic equilibrium during the analysis. In the case of these simulations, the internal forces generated by material stresses are counterbalanced by the applied external forces, which are represented as displacements.

In addition, the Ansys software, when carrying out simulations of non-linear structural behavior, uses the Newton–Raphson method to solve the system of non-linear equations associated with the analysis. This method is used in non-linear problems due to its efficiency in guaranteeing convergence [24]. The procedure involves an iterative approximation of the solution, where at each iteration the system of differential equations is linearized around an estimated equilibrium point, and the internal and external forces are adjusted. During each step of the simulation, Ansys recalculates the deviation between the applied forces and the internal reactions, making successive corrections until the difference is small enough to reach convergence. The exact solution to the problem occurs when equilibrium between the forces is reached [25].

After reaching convergence using the Newton–Raphson method, the system is solved using numerical methods for solving systems of linear equations, such as matrix decomposition or iterative methods, depending on the complexity of the model and the number of variables involved. This process ensures that the responses generated, such as displacements and stresses, are calculated accurately and conform to the conditions imposed in the model, providing reliable results that are consistent with the simulated physical reality.

The cube and the specimen simulations serve distinct but complementary purposes in this study. The simulation of the cube is primarily aimed at determining the most suitable constitutive relationship for the material, given its simplicity and efficiency in simulation. This approach allows for an effective comparison of different material models and the calibration of parameters to closely match experimental data. On the other hand, the simulation of the specimen is performed to validate the robustness and reliability of the selected constitutive model. By demonstrating that the adopted model accurately represents the material’s behavior under realistic conditions, it ensures that the simulation framework can effectively predict the performance of the material in practical applications. This dual approach—simplifying with the cube and verifying with the specimen—provides a comprehensive understanding of the material’s mechanical response and strengthens the confidence in its use for design and application in soft grippers or similar devices.

In Ansys, for both the unit cube and the specimen, a high-density mesh with automatic resolution and level 3 refinement (maximum) was applied. This configuration ensures that the mesh quality is balanced, providing sufficient resolution to accurately capture the material’s behavior in the regions of interest without compromising computational efficiency. The element size was standardized and controlled by the program, with adaptive behavior and quick transitions between mesh regions to ensure precision and numerical convergence.

**Figure 16:**
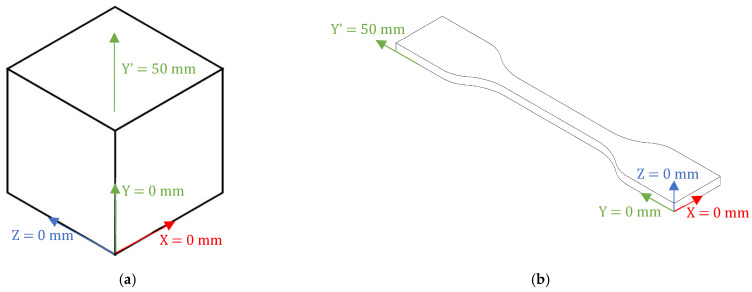
Schematic representation of Boundary conditions for: (**a**) Unit Cube; (**b**) Specimen.

For the unit cube, quadratic hexahedral elements (Hex20) were automatically selected by the program. This type of element is used for regular geometries and performs well in terms of accuracy and computational efficiency. The cube’s mesh consists of 8281 nodes (2197 corner nodes and 6084 intermediate nodes) and 1728 elements, with 3 degrees of freedom per node. The unit cube schematic representation of boundary conditions is shown in Figure 16a. Due to the geometry of the adopted specimen, second-order tetrahedral elements (Tet10), with 3 degrees of freedom per node, were used in the discretization. In fact, these types of elements are known to be particularly suitable for meshes with complex geometries. In terms of mesh properties, the specimens were discretized into 6217 elements with 12,602 nodes in total (2185 corner nodes and 10,417 intermediate nodes). The region of greatest interest is the gauge length section, and an example of the schematic representation of the adopted boundary conditions is shown in Figure 16b.

**Figure 17:**
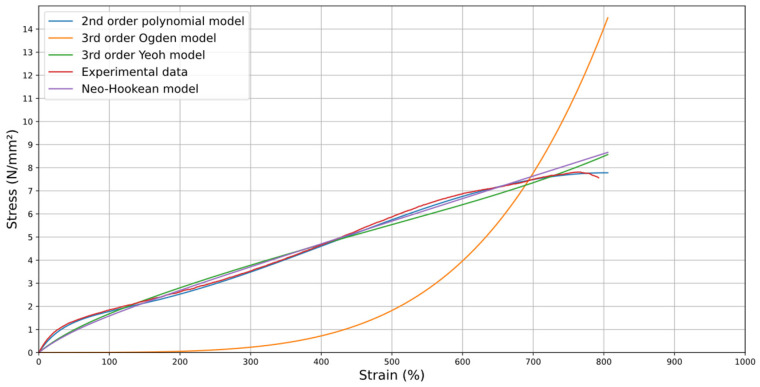
Constitutive models and average experimental test data for TPU 60A with quasi-isotropic orientation at v = 20 mm/min.

For the unit cube model, four constitutive models were selected for testing: Neo-Hookean, 2nd Order Polynomial, 3rd Order Yeoh, and 3rd Order Ogden. These models were identified for their ability to accurately represent the non-linear elastic behavior of flexible gripper materials. The stress–strain curve of the four constitutive models considered for selection is shown in Figure 17.

By analyzing Figure 17, it becomes evident that the 2nd Order Polynomial model is the most promising among the evaluated options. Additionally, the coefficient of determination ($R^{2}$) values for all the tested mathematical models, as presented in Table 4, further support this conclusion.

Thus, the 2nd Order Polynomial model has the highest *R*^2^ value, demonstrating the best fit to the experimental data. This strong correlation supports our selection of this model for accurately representing the mechanical behavior of TPU 60A in the simulations. And, to validate its accuracy, a coefficient of determination ($R^{2}$) value of 0.9986 was achieved between the experimental data and the model, demonstrating that it captures nearly all the variability in the data and provides a highly reliable representation of the stress–strain behavior. Additionally, as highlighted by Jaiswal et al. [26], recent studies have shown that this model effectively represents the mechanical response of similar materials, accurately capturing viscoelastic deformations under varying loading conditions. This has been consistently validated through multiple computational simulations and comparisons with experimental results.

**Figure 18:**
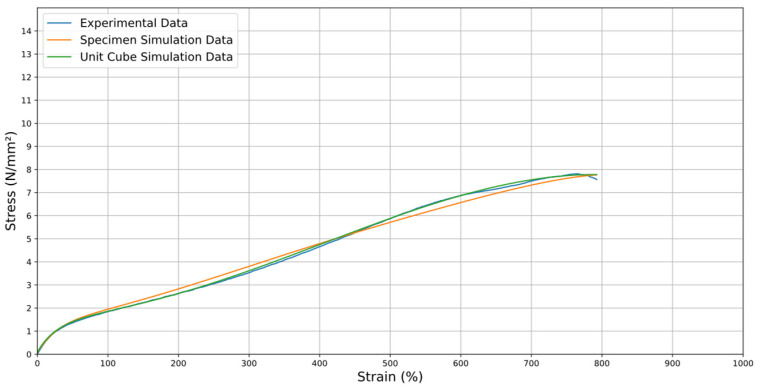
Comparison between the average stresses of the experimental data, the simulation data of the specimens, and for the unit cube.

Figure 18 shows the comparison between the stress observed in the experimental study and the simulations of the unit cube and the specimen. The close correspondence between the curves confirms that the constitutive model is appropriate for predicting the material’s mechanical behavior, ensuring the model’s reliability.

As a result, the graph in Figure 18 was generated, showing three curves, one for the actual average stress (in blue), other for the interpolated stress that occurred in the computational simulation for the specimen (in orange), and the computational simulation of the unit cube data (in green).

According to Ansys [27], the equation for the 2nd Order Polynomial model is as follows:

(1) $$\psi_{P}=\sum_{m+n=1}^{N}C_{mn}\times {\left(\bar{I_{1}}-3\right)}^{m}\times {\left(\bar{I_{2}}-3\right)}^{n}+\sum_{k=1}^{N}\frac{1}{d_{k}}\times {\left(J-1\right)}^{2k},\;\;N=2$$

where $\psi_{P}$ = strain-energy potential; $C_{mn}$ = Material constants; $\bar{I_{1}}$ is the first deviated principal invariant; $\bar{I_{2}}$ is the second invariant of the isochoric left or right Cauchy–Green deformation tensor; *d_k_* = material incompressibility parameters; J = determinant of the elastic deformation gradient F; N = material constant. The initial shear modulus is defined as Equation (2):

(2) $$\mu_{P}=2\times \left(C_{10}+C_{01}\right)$$

and the initial bulk modulus for this case is defined as Equation (3):

(3) $$K_{P}=\frac{2}{d_{k}}$$

Material constants are used to define the parameters of the 2nd Order Polynomial constitutive model, which describes the material’s mechanical behavior under stress. Together, these constants calibrate the 2nd Order Polynomial model to match the experimental data, ensuring the simulation accurately reflects the mechanical behavior of the material under various loading scenarios. These values are listed in Table 5.

It is important to note that the coefficients used in the constitutive models are generated automatically by Ansys in the materials tool, based on the experimental data entered. The software accurately adjusts the data to calculate the necessary coefficients. It is then necessary to select the curve that best represents the material’s constitutive behavior, ensuring equivalence with the tensile tests carried out.

Thus, the constitutive model that best represents the curve is defined using Equation (4).

(4) $$y=-7\times 10^{-6}x^{2}+0.018x+0.7173$$

Equation (4) describes the relationship between stress and strain in the material as predicted by the model. This equation allows for interpolation and extrapolation of data, enabling the prediction of material behavior under different strain conditions. Moreover, it provides a mathematical representation that can be directly implemented in computational simulations, ensuring consistency and accuracy when comparing experimental and simulated results. The high $R^{2}$ value associated with this equation further demonstrates its reliability in capturing the material’s mechanical response.

**Figure 19:**
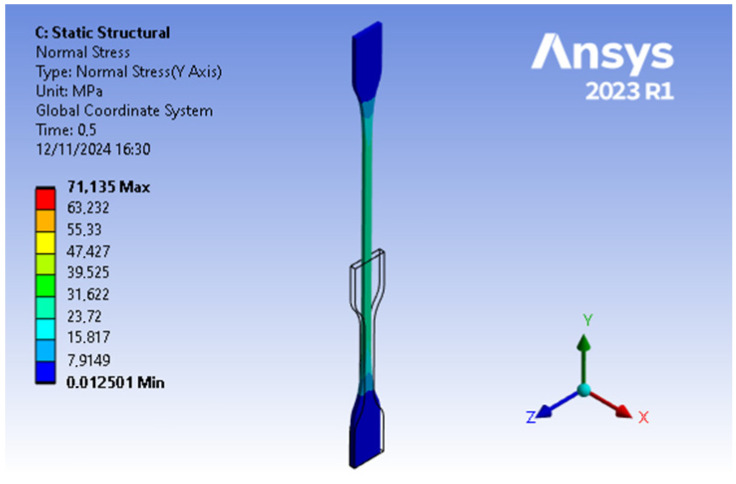
Specimen during a simulation at 0.5 s with scale of 1:2.

It is important to note that, although the software automatically selected different types of elements for the cube and the specimen, the simulation results remain valid. In the case of the cube, Hex20 elements were used, while the specimen was modeled with Tet10 elements. This is because both types of elements, when properly configured and refined, can capture the mechanical behavior of the material in the regions of interest. Figure 19 displays the distribution of normal stress values during the numerical test at 0.5 s, as visualized in the Ansys software.

The stress–strain diagrams presented in Figure 18 show three distinct datasets: (1) the results from the elongation of the unit cube in numerical simulations, (2) the results from the elongation of the ISO specimen in numerical simulations, and (3) the tensile test results of ISO specimens obtained experimentally.

The unit cube simulation was conducted to identify the most suitable constitutive relationship for the material, leveraging its simplicity and simulation efficiency. This approach facilitated an effective comparison of different material models and enabled the calibration of parameters to closely match experimental data. By using the stress–strain data obtained experimentally, the numerical simulations of the unit cube established a solid foundation for selecting the most appropriate constitutive model. Once calibrated, this model was applied to simulate the tensile behavior of the ISO specimens, enabling a direct comparison with the experimentally obtained tensile test data. This process successfully validated the constitutive model’s ability to accurately predict the material’s mechanical response under practical conditions.

On the other hand, the numerical simulation with the specimen is conducted to validate the robustness and reliability of the selected constitutive model. By demonstrating that the adopted model accurately represents the material’s behavior under realistic conditions, it ensures that the simulation framework can effectively predict the material’s performance in practical applications. This dual approach—simplifying with the unit cube and verifying with the specimen—provides a comprehensive understanding of the material’s mechanical response and reinforces confidence in its use for different designs, such as the application of soft grippers or similar devices.

In Figure 18, the stress–strain curves compare the results from the numerical simulation of the ISO specimen, unit cube, and the experimental tensile test data. It is important to note that the stress values in this figure correspond to the averaged global response of the material and are not directly comparable to the localized stress values shown in Figure 19, which shows the distribution of normal stress during the numerical tensile test of the ISO specimen at 0.5 s, as simulated in Ansys software. The legend in this figure displays the range of stress values, with a maximum value of 71.135 MPa at the specified moment in the simulation. This value represents the localized stress at specific nodes and is derived from the computational model.

The $R^{2}$ value for the comparison between the cube data and the specimen data is 0.9927. Also, the $R^{2}$ value was calculated for the comparison between the cube data and the experimental data, which is 0.9714, and the $R^{2}$ value for the comparison between the specimen data and the experimental data, which is 0.9892. These results indicate a good fit and high agreement between the two datasets. Having high $R^{2}$ value confirms that the simulation model is well-calibrated and effectively captures the material’s mechanical behavior. Furthermore, it validates that the material properties defined for the simulation align closely with the experimental observations, ensuring the reliability and accuracy of the simulation in predicting the material’s stress–strain response.

Then, in this simulation, the maximum force reached was 62.132 N. All the data was divided by the initial cross-section area (*A*_0_ = 8.00 mm^2^), reaching a maximum stress of 7.81 N/mm^2^ for the experimental values and a maximum stress value of 7.77 N/mm^2^ for the simulation results. This means that although the specimen breaks at approximately 372.50 mm of displacement in practice, the maximum force reached occurs at approximately 360.50 mm, approximately 13.00 mm after that predicted in the simulation. The simulation is experimentally validated, with only an absolute stress error of 0.04 N/mm^2^, corresponding to a relative error of approximately 0.5%.

To validate the model, the relative errors of both force and displacement were calculated. The Relative Error of the Maximum Stress ($E_{rSm}$) is given by Equation (5):

(5) $$E_{rSm}\left(\%\right)=\left(\frac{7.81-7.77}{7.77}\right)\times 100\;E_{rSm}\left(\%\right)≅0.51\%$$

The Relative Error of the Displacement where the Stress is Maximum ($E_{rDm}$) is given by Equation (6):

(6) $$E_{rDm}\left(\%\right)=\left(\frac{360.54-346.00}{360.54}\right)\times 100\;E_{rDm}\left(\%\right)≅4.03\%$$

In addition, the force error was calculated at the exact point of 346 mm, and it was first necessary to carry out a linear interpolation of the average of the realized force, which is given by Equation (7):

(7) $$y=y_{1}+\frac{\left(x-x_{1}\right)}{\left(x_{2}-x_{1}\right)}\times \left(y_{2}-y_{1}\right)$$

where, according to the data taken from the average stress × strain, the following values are approximated to 15 decimal places: $x_{1}$ = 345.61 mm; $y_{1}$ = $\frac{61.57}{8.00}$ N/mm^2^; $x_{2}$ = 346.35 mm; $y_{2}$ = $\frac{61.61}{8.00}$ N/mm^2^; x = 346.00 mm. This is given by Equation (8):

(8) $$y=\frac{61.57}{8.00}+\frac{\left(346.00-345.61\right)}{\left(346.35-345.61\right)}\times \left(\frac{61.61\;-61.57}{8.00}\right)\;y≅7.70\;N/{mm}^{2}$$

Then, the relative error where the displacement of 346 mm occurs is given by Equation (9):

(9) $$E_{rd}\left(\%\right)=\left|\frac{61.59-62.13}{61.59}\right|\times 100\;E_{rd}\left(\%\right)≅\;0.88\%$$

In the Ansys software, the TPU 60A material was created in the Engineering Data section and the 2nd Order Polynomial model was added. In the properties table, it was noted that the material has a density (ρ) of 1070 kg/m^3^ and that the tests were carried out at room temperature (*T* = 22 °C). The average displacement (mm/mm) and stress (N/mm^2^) values for 500 points of the quasi-isotropic specimens at v = 20 mm/min were added to the uniaxial test section. The curve was adjusted and the results copied to the creation of the material.

Thus, it was noticed that exploring different constitutive models helps to accurately capture the complex mechanical behavior of materials under various loading conditions. Models such as the 2nd order polynomial, 3rd order Ogden, Neo-Hookean, and 3rd order Yeoh provide distinct approaches to describing the stress–strain relationship, each suited to specific material characteristics. Comparing these models against experimental data helps identify the most accurate representation, optimize simulation performance, and understand the limitations of each model. This ensures reliable predictions and better alignment with the material’s real-world behavior.

### 4.3. Real-World Applications and Challenges of TPU 60A

TPU 60A has proven its utility in various real-world applications, particularly in industries requiring flexibility and adaptability. In soft robotics, it serves as a material of choice for grippers due to its capacity to handle delicate objects without causing damage, as observed by Gaafar et al. [28], who introduced the design for a JD-printed soft pneumatic bending actuator, material characterization, and 3D printing parameter optimization of flexible material with 60A shore hardness. This makes this application valuable in agriculture for harvesting fruits and vegetables and in the food industry for manipulating fragile items during packaging processes. Similarly, TPU 60A is used in the manufacturing of wearable medical devices and assisted technologies, since its elasticity ensures comfort and functionality. Despite these advantages, practical applications face some challenges, such as material fatigue under cyclical loads, sensitivity to environmental factors like humidity, and limitations in large-scale manufacturing. Addressing these challenges requires further material optimization, particularly in terms of humidity control during processing and post-treatment methods to enhance durability [28,29]. While this study offers significant insights into the mechanical properties of TPU 60A, some limitations should be acknowledged. The research primarily focused on uniaxial tensile tests and specific printing orientations, which may not fully capture the material’s behavior under real-world stress conditions. Future studies could explore biaxial or multiaxial stress conditions to better understand its performance in practical applications. Additionally, the long-term behavior of TPU 60A under cyclic loading remains unexplored, which is critical for applications involving repetitive mechanical stress. To address these gaps, future research should incorporate fatigue testing to develop advanced constitutive models [29]. Further, the development of tailored G-code algorithms for graded material deposition could minimize stress concentrations and enhance structural integrity. Exploring hybrid materials or composites involving TPU 60A also presents promising research paths for achieving superior performance in demanding applications. Recent studies, for example, demonstrate improvements in mechanical properties and environmental resistance through the addition of nanofillers like carbon nanotubes or graphene further underscore the potential of such advancements [30].

## 5. Conclusions

It is evident in the characterization of TPU 60A material, carried out through tensile tests for the orientations of 0°, 45° and −45°, 90°, and quasi-isotropic, that both the printing orientation and test speed have a significant impact on the material’s performance. These factors directly influence key mechanical properties, including force, stress, strain, and the overall deformation behavior. While some orientations exhibit greater resistance, others prove to be less effective for these purposes. Among the tested orientations, the quasi-isotropic configuration was found to be the most suitable for representing the material’s behavior in a balanced manner.

Despite the TPU 60A filament having well-defined properties provided by the manufacturer, such as an elongation at break of 950% [12], factors related to the printing process can significantly alter these characteristics. Variables such as printing orientation, environmental humidity, and the geometric design of the printed structure significantly affect the final mechanical properties of the material, including its strength, ductility, and strain capacity. These modifications can lead to deviations from the nominal values, highlighting the importance of carefully considering these factors in the context of application. This ensures that the desired performance is achieved and aligns with the requirements of the specific use case. Also, in the present study, the material preparation and printing process were calibrated based on the manufacturer’s recommendations, provided in the datasheet for Filaflex TPU 60A. Key parameters such as nozzle temperature (230 °C), infill density (100%), and layer height (0.25 mm) were set in accordance with these guidelines to ensure consistency with standardized practices, but future work could explore the interaction of these parameters to build upon the findings presented here.

From the average data of the five valid tensile tests, the uniaxial behavior required to create the TPU 60A material library in the Ansys software was obtained. Using the second-degree polynomial method, these data were used to calibrate and validate the material model through two approaches: first, a numerical simulation of a unit cube to evaluate and refine the constitutive model parameters, and second, a simulation of the tensile test using ISO specimens to validate the model against experimental results. It is important to note that the unit cube test was not conducted experimentally; it served as a numerical tool for calibration, while the experimental tensile tests provided the primary validation of the material model.

Reducing the presence of voids in printed specimens is important for enhancing mechanical properties and reliability, and controlling filament humidity significantly decreases void formation by optimizing the printing parameters such as extrusion settings, nozzle diameter, print paths, and infill patterns. Fine-tuning these factors leads to stronger, more consistent specimens, resulting in improved performance of the TPU 60A. In future work, additional experimental data focusing on precise measurements of void density under varying humidity levels and their direct impact on mechanical properties such as tensile strength and elongation at break should be analyzed.

The findings presented in this study provide a strong foundation for the use of TPU 60A in soft robotics applications, particularly in the design and optimization of soft robotic grippers. To further establish the practical relevance of these results, it is recommended for future work to conduct practical tests using a robotic arm equipped with TPU 60A grippers. Such tests would allow for evaluating the grippers’ performance in real-world scenarios, including their adaptability to delicate or irregularly shaped objects. Additionally, laboratory experiments designed to test the grasping ability and effectiveness of the grippers under various conditions would provide deeper insights into their practical capabilities. These tests would demonstrate how the material’s mechanical properties translate into functional advantages, ensuring a clearer alignment between the material characteristics and their application in soft robotics.

This work offers significant contributions to the development of elastomer grippers, as well as providing valuable insights for future projects. Among the suggestions for future work, one key recommendation is the manual development of a G-code that allows for varying the material deposition thickness during the printing process, resulting in a variable thickness. This approach reduces the formation of defects in the material, promoting stress relief and significantly reducing stress concentrations. Consequently, it contributes to the production of a more uniform part, yielding more consistent and reliable results.

The inclusion of biaxial tests for a more comprehensive and accurate characterization of elastomeric materials, such as TPU, is also suggested. By applying stresses in two directions simultaneously, these tests allow for a more reliable capture of the materials’ real behavior, especially in applications involving complex deformations, as is the case with soft grippers. This would enable the acquisition of richer experimental data, which can be used to refine computational models, improve the simulation of grippers under multiple stress conditions, and optimize performance in real-world scenarios.

## Figures and Tables

**Table 1:** Main specifications used in Cura software (version 5.0) for TPU 60A.

Specification	Value	Specification	Value
Nozzle diameter	0.6 mm	Line width	0.58 mm
Layer height	0.25 mm	Printing temperature	230 °C
Infill density	100%	Printing speed	20 mm/s
Infill pattern	Lines	Retraction distance	4.5 mm
Flow	200%	Retraction speed	25 mm/s
Number of layers	16–ISO-527 8–ISO 37	Infill line directions	[0°; 45°, −45°; 90°; Quasi-Isotropic]

**Table 2:** Summary of the characteristics of the TPU 60A characterization tests.

Tests	Quasi-Isotropic	0°	45° and −45°	90°
Number of valid specimens	13	10	10	10
Nomenclature of the specimens	QI-02-01; QI-02-02; QI-02-03; QI-20-01; QI-20-02; QI-20-03; QI-20-04; QI-20-05; QI-200-01; QI-200-02; QI-200-03; QI-200-04; QI-200-05;	O-20-01; O-20-02; O-20-03; O-20-04; O-20-05; O-200-01; O-200-02; O-200-03; O-200-04; O-200-05;	F-20-01; F-20-02; F-20-03; F-20-04; F-20-05; F-200-01; F-200-02; F-200-03; F-200-04; F-200-05;	N-20-01; N-20-02; N-20-03; N-20-04; N-20-05; N-200-01; N-200-02; N-200-03; N-200-04; N-200-05;
Test speeds (v) tested	2 mm/min; 20 mm/min;	20 mm/min;	20 mm/min;	20 mm/min;
	200 mm/min;	200 mm/min;	200 mm/min;	200 mm/min;

**Table 3:** Comparison between the percentage of voids area inside the samples using ImageJ software.

Samples	Voids Area
QI-60A-HUM (+37%)	12.13%
QI-60A-NONHUM (±22%)	2.80%
O-60A-HUM (+37%)	24.90%
O-60A-NONHUM (±22%)	3.87%
F-60A-HUM (+37%)	9.94%
F-60A- NONHUM (±22%)	3.76%
N-60A-HUM (+37%)	15.12%
N-60A-NONHUM (±22%)	2.98%

**Table 4:** Coefficient of determination (*R*²) values for different material models.

Models	$\mathrm{Values}\;\mathrm{of}\;\mathrm{Coefficient}\;\mathrm{of}\;\mathrm{Determination}\;(R^{2})$
2nd Order Polynomial model	0.9993
3rd Order Yeoh model	0.9843
3rd Order Ogden model	−0.8049
Neo-Hookean model	0.9857

**Table 5:** Material constants (*C*_*mn*_) and incompressibility parameters (*d*_1_, *d*_2_) of the 2nd Order Polynomial constitutive model, as related to Equation (1).

Constants	Value
Material constant 10 [Pa]	$2.6643\times 10^{5}$
Material constant 01 [Pa]	$6.6007\times 10^{5}$
Material constant 20 [Pa]	−7852.1
Material constant 11 [Pa]	80,523
Material constant 02 [Pa]	$-1.6973\times 10^{5}$
Incompressibility parameter *d*_1_ [Pa^−1^]	0
Incompressibility parameter *d*_2_ [Pa^−1^]	0

## Data Availability

The original contributions presented in the study are included in the article, further inquiries can be directed to the corresponding author.
